# FTIR Spectroscopy Study of the Secondary Structure Changes in Human Serum Albumin and Trypsin under Neutral Salts

**DOI:** 10.3390/biom10040606

**Published:** 2020-04-14

**Authors:** Dmitrii Usoltsev, Vera Sitnikova, Andrey Kajava, Mayya Uspenskaya

**Affiliations:** 1 Department of Applied Optics, ITMO University, St.-Petersburg 197101, Russia; dmitriy.usolcev.97@mail.ru (D.U.); mv_uspenskaya@mail.ru (M.U.); 2 International Research Institute of Bioengineering, ITMO University, St.-Petersburg 197101, Russia; andrey.kajava@crbm.cnrs.fr; 3 Centre de Recherche en Biologie cellulaire de Montpellier (CRBM), UMR 5237 CNRS, Université de Montpellier 1919 Route de Mende, CEDEX 5, 34293 Montpellier, France

**Keywords:** protein denaturation, FTIR spectroscopy, second derivative method, secondary structure, neutral salts, aggregates

## Abstract

The effect of neutral salts on protein conformation was first analyzed by Hofmeister in 1888, however, even today this phenomenon is not completely understood. To clarify this effect, we studied changes in the secondary structure of two proteins: human serum albumin with predominantly α-helical structure and porcine pancreas β-trypsin with the typical β-structural arrangement in aqueous solutions of neutral salts (KSCN, KCl, (NH_4_)_2_SO_4_). The changes in the secondary structure were studied at 23 °C and 80 °C by using the second derivative deconvolution method of the IR spectra. Our results demonstrated that the ability of the salts to stabilize/destabilize these two proteins correlates with the Hofmeister series of ions. At the same time, some exceptions were also observed. The destabilization of the native structures of both α-helical albumin and β-structural trypsin upon interaction with neutral salts leads to the formation of intermolecular β-sheets typical for amyloid fibrils or amorphous aggregates. Thus, our quantitative FTIR-spectroscopy analysis allowed us to further clarify the mechanisms and complexity of the neutral salt actions on protein structures which may lead to strategies preventing unwelcome misfolding of proteins.

## 1. Introduction

The effect of neutral salts on protein structures and folding-unfolding events is of particular interest because neutrals salts are widely used in molecular biology to modulate the stability and association of proteins, as well as their salting-out and crystallization [1,2]. They are also used in biotechnology to isolate expressed recombinant proteins [3] and to control enzyme activities [4,5]. The concept of ion specificity in salt-protein systems comes from Hofmeister’s works of the 1880s. He classified the ion series depending on the salting-out effect (later called the Hofmeister series) [1,2,4]. The modern version of the anionic and cationic Hofmeister series are SO_4_^2−^, HPO_4_^2−^, acetate, citrate, Cl^−^, NO_3_^−^, ClO_3_^−^, I^−^, ClO_4_^−^, SCN, and NH_4_^+^, K^+^, Na^+^, Li^+^, Mg^2+^, Ca^2+^, guanidinium, respectively [6]. These series’ rank the power of the ion effect on the solubility of proteins and their stability.

In general, aqueous solutions of neutral salts have two effects on proteins. The first effect does not depend on the nature of the ion. This nonspecific effect reduces electrostatic intramolecular repulsion and stabilizes the macromolecule. It is usually observed at a low ionic strength of the solution up to about one-tenth of ionic strength [1,7]. The second effect, called the specific lyotropic effect, is observed at higher concentrations of neutral salts. This effect manifests in the stabilization/destabilization of the native protein structure, mainly by changing the structure of water and the energy at the solvent-protein interface [1,5,8,9]. For example, by using the Poisson–Boltzmann approach, it was shown that this specific effect is responsible for the stabilization of α-helices [10]. Molecular dynamic simulations of model α-helical peptides supported these observations [11]. One of the founders of the theory of specific ion-protein interactions, Kim Collins, introduced notions of “chaotropic” ions as ions with a low charge density and are, therefore, poorly hydrated. They have a negative Jones–Dole viscosity B coefficient [12], as opposed to “kosmotropic” ions, with a high charge density and strong hydration. The kosmotropic ions have a positive Jones–Dole viscosity B coefficient [12]. This is consistent with FTIR spectroscopy data about the effect of the neutral salts in aqueous solution on hydrogen bonds (H-bonds). For example, Der et al. showed that chaotropic anions such as thiocyanate and perchlorate weaken intermolecular H-bonds in comparison with pure water. At the same time, kosmotropic anions, such as fluoride and acetate, increase the number of intermolecular H-bonds [13]. The hydration of ions underlies its “law of matching water affinities” (LMWA), which in many cases explains the effects of neutral salts on proteins [14,15,16].

To explain the interactions of neutral salts with proteins, Kim Collins proposed three interdependent layers in the interfacial space between protein and water. The first layer directly contacts the protein surface, the second transition layer adjoins to the first and the third contains bulk water. In the absence of salt, the first (and partially second) water layer is predominantly involved in protein hydration; when salt with a kosmotropic ion is added, the second layer will preferably participate in hydration of the salt ion rather than protein. In such conditions, the protein folds more compactly and reduces the interaction area with the solvent [7,17,18]. Along this line, Pace et al. indicated, according to experimental solubility results and based on the free transfer energy, that globular proteins are more stable in non-polar solvents and even more stable in a vacuum [19]. In the case of a chaotropic ion, the transition layer will preferably participate in protein hydration. There is an unfolding of the native state, partially due to the direct adsorption of chaotropic ions on the protein surface, while kosmotropic ions stay in solution [7,17,18,20]. Thus, kosmotropes increase and chaotropes decrease protein–water interfacial tension, making the protein–water interfaces more “hydrophobic” and “hydrophilic,” respectively [13]. Several spectrometric and thermodynamic experiments, molecular modeling simulations, and applications of LMWA revealed the basic principles of the interaction of the ions with proteins [6,21,22,23,24,25].

By summarizing the described information above [7,16,17,18,20,21,22], we can distinguish the following tendencies:

- the adsorption of ions on the protein surface destabilizes the protein structure,
- the ions are adsorbed if they have an affinity for the protein surface,
- the affinity for the protein surface can be predicted using LMWA.

Although Collins’ theory tried to explain all salt-protein interactions, there are many exceptions from these rules. For example, in the inverse Hofmeister series, ions that fall out of the ion series are frequent [1]. Previous studies showed that chaotropes destabilize the native structure of proteins with positive interfacial tension, and have the opposite effect on proteins with negative interfacial tension [13]. Lysozyme is known to be salted out of solution according to the direct Hofmeister series at high ionic strength and basic pH values but follows the inverse Hofmeister series at low ionic strength and acidic pH values [26]. Lopez-Arenas et al. showed that, regardless of the type of neutral salt, the effect of electrostatic screening stabilizes the transition state of chymopapain over the native state [8]. It was also shown that all anions of the Hofmeister series destabilize the native state of α-chymotrypsin [14]. The structural state of denatured proteins under the influence of destabilizing ions remains unclear and is often interpreted as a compact dry molten globule [27]. Usually, conformational rearrangements that accompany protein destabilization are not investigated. Only in some studies, the change in the α-helicity of the protein structure is recorded as a consequence of structural destabilization [28,29]. Thus, due to competing effects non-polar and polar solvation at protein surfaces, the detailed molecular mechanism of the effect of neutral salts on protein structures is still a subject of intense investigation [30]. Thus, the accumulation of experimental studies of particular protein-salt systems is needed to improve the theory.

Previously, by using FTIR spectroscopy, we have shown that a second derivative method was suitable for detecting and quantification the small changes in the secondary structure of the HSA depending on different denaturing agents [31]. In this work by using this FTIR spectroscopy approach, we studied the effects of neutral salts KSCN (strong chaotrope), KCl (weak chaotrope), and (NH_4_)_2_SO_4_ (strong kosmotrope) on the secondary structure and thermostability of two different proteins. One of them was human serum albumin (HSA), a typical α-helical globular protein with a molecular weight of 66.5 kD and a pI value of 4.8. The other was porcine pancreatic trypsin, a typical β-structural protein (23.4 kDa) with six intramolecular disulfide bridges and a pI value near 10.8.

## 2. Materials and Methods

Human serum albumin (HSA) was used in the form of 20% wt. aqueous solution for infusion (Microgen, Russia) without additional purification. Porcine pancreatic trypsin was used in the form of 40% wt. aqueous solution (Biolot, Russia). Five ions were selected to evaluate their effect on the secondary structure of HSA and trypsin. Their typical effects on proteins are shown in Table 1.

Protein-salt solutions were prepared by mixing 150 μL of a 20% wt. ready-for-use aqueous HSA solution (40% wt. trypsin) with 150 μL of the KCl, KSCN, (NH_4_)_2_SO_4_ solution in distilled water, which were presented in a series: 0.05 M; 0.2 M; 0.5 M; 1 M; 1.5 M; 2 M for each salt, respectively. Protein–salt solutions for thermal denaturation were prepared similarly. Denaturation was carried out in a thermostat, incubating the solution for 10 min at 80 °C. The FTIR spectrum measurement at a high temperature can provide us with additional information about the effect of neutral salt on protein stabilization. The reference solution for thermal denaturation was a 10% wt. aqueous solution of HSA and 20% wt. aqueous solution of trypsin, incubated for 10 min at 80 °C.

IR spectra of attenuated total internal reflection (ATR) of the samples were recorded in the range of 4000–600 cm^–1^ on a Bruker Tensor 37 FTIR spectrophotometer using an ATR accessory (diamond coated ZnSe crystal) with a spectral resolution of 2 cm^–1^ and averaging on 164 scans. The solvent spectrum has been subtracted iteratively until a straight baseline is obtained in the spectral region of 2000–1750 cm^−1^.

The method of deconvolution of the second derivative of the IR spectrum was used to track the changes in the secondary structure of HSA and trypsin depending on various factors.

**Figure 1:**
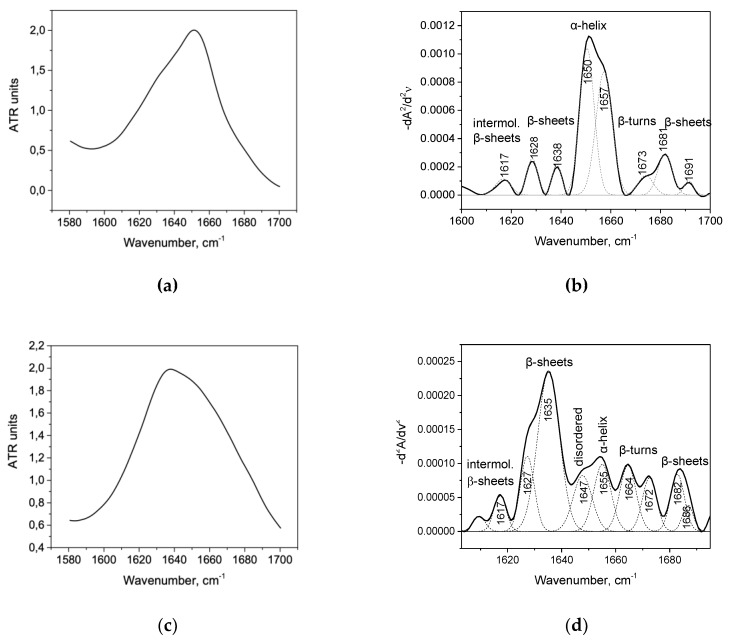
The Amide I band (**a**) and its second derivative deconvolution (**b**) of native human serum albumin (HSA) in 10% wt. aqueous solution. The Amide I band (**c**) and its second derivative deconvolution (**d**) of native trypsin 20% wt. aqueous solution.

The protein spectra used for analyses were obtained by subtracting the spectra of an aqueous solution using Opus 7.0 software. The secondary structure was determined by deconvolution of the second derivative of the Amide I band in the OriginPro 2015 software (Figure 1, Figures S1–S6). All second derivatives have been multiplied by -1 to correspond to the curves that were published in the previous works. The procedure for calculating the secondary structure of proteins using the deconvolution of the second derivative of the spectrum is described in detail in work [32].

## 3. Results and Discussion

### 3.1. Spectral Studies of Native Protein Structures

According to the previous spectral studies of globular and fibrillar proteins [33], the Amide I area and peaks obtained from its second derivative deconvolution correspond to the following secondary structures (Table 2).

Albumin peaks at 1650 and 1657 cm^−1^ refer to 60.3% of the total area of the second derivative (Figure 1b), and peak 1655 cm^−1^ of trypsin refer to 11.2%, which agrees with the data obtained from the DSSP, STRIDE, and the secondary structure assigned by authors of the X-ray structures PDB codes 1AO6 (HSA) and 1FMG porcine beta trypsin) [34,35,36,37,38,39] (Table 3). HSA proteins do not have a maximum at the second derivative deconvolution curves that correspond to a random coil conformation defined by the range of 1640-1649 cm^−1^ [33], which is also consistent with the previous FTIR studies [33,40,41,42]. In addition, the maxima of the second derivative (1628 cm^−1^, 1638 cm^−1^, and 1691 cm^−1^) characterize the β-sheet content, referring to the study of serum albumin [41]. The maxima of 1627 cm^−1^ and 1635 cm^−1^ of trypsin refer to 46.9% of the total area (Figure 1d), which is consistent with the X-ray data (Table 3) and was observed previously by FTIR [42].

### 3.2. Salt-Induced Transition

**Figure 2:**
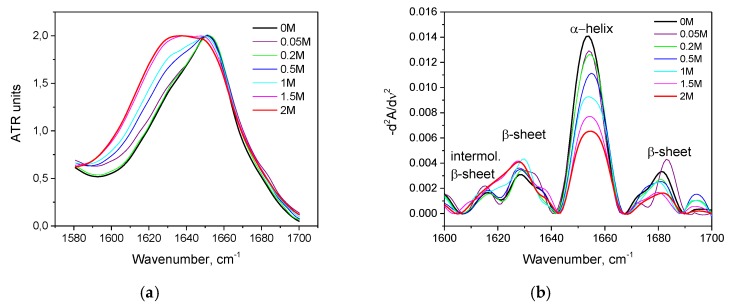
Changes in the Amide I band (**a**) and changes in the second derivative of the Amide I band (**b**) of HSA in 10% wt. aqueous solution at 23 °C with various concentrations of KSCN.

**Figure 3:**
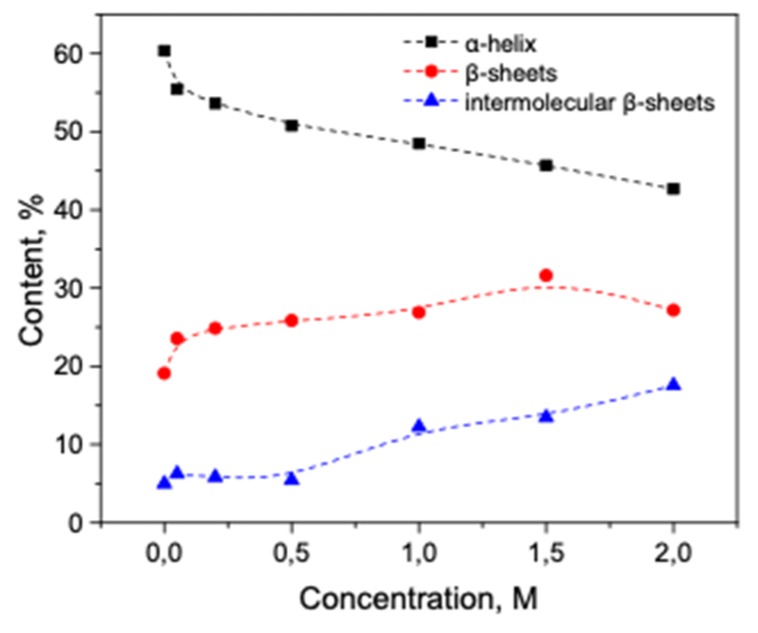
Potassium thiocyanate-induced conformational transition of HSA at 23 °C. Protein concentration was 10% wt.

**Figure A1:**
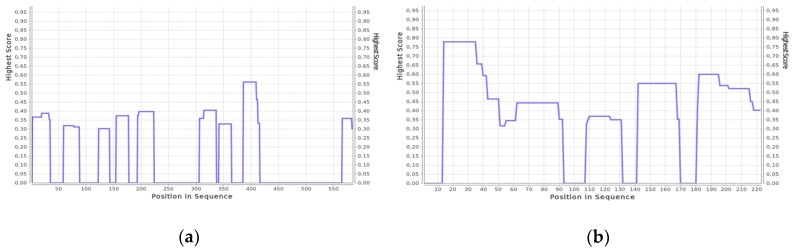
Prediction of amyloidogenic regions (denoted by blue contours) in the unfolded HSA (**a**) and trypsin (**b**) by using the ArchCandy program.

As seen in Figure 2a, with an increase in the concentration of KSCN, there is a noticeable change in the Amide I band of HSA in the regions 1620–1640 cm^–1^ and 1650–1660 cm^–1^, corresponding to β-sheets and α-helix, respectively. On the second derivative curve (Figure 2b), one can see a gradual decrease in the contribution of the α-helix region and an increase in the contribution of the β-sheets area (Figure 3). In addition, the deconvolution of the second derivatives of the series shows that at a concentration of 1 M KSCN and above, the proportion of the region 1615–1625 cm^−1^ corresponding to the intermolecular β-sheets increases significantly (Figure 3). In the 2 M KSCN solution, the proportion of intermolecular β-sheets is 17.5%, which is 3.5 times greater than that of the native form. In addition, it can be noted that, at a concentration of 1.5 M, the maximum content of β-sheets is reached (32%), and at 2 M their content slightly decreases probably due to a significant increase in intermolecular β-sheets (Figure 3). Thus, we observe conformational transitions of α-helix→ intra- and intermolecular β-sheets. It is known that intermolecular β-sheets are frequently linked to the formation of amyloid aggregates. Indeed, our analysis of the HSA amino acid sequence by using ArchCandy program [43] revealed that a significant part of this protein has amyloidogenic regions, which has the potential to form intermolecular cross-β-amyloids when unfolded (Figure A1).

**Figure 4:**
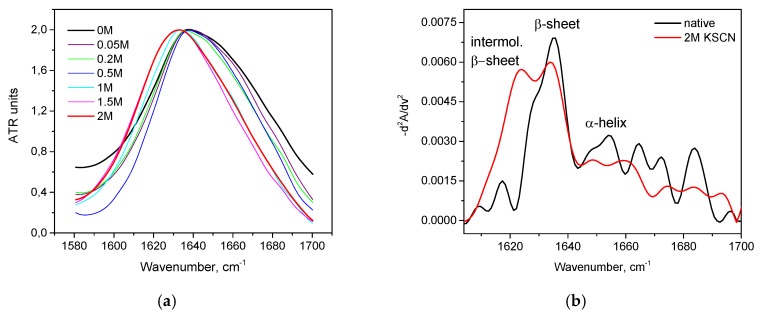
Changes in the character of the Amide I band (**a**) and changes in the second derivative of the Amide I band (**b**) of trypsin in 20% wt. aqueous solution at 23 °C with various concentrations of KSCN. Only the most representative curves are shown for the sake of clearness.

**Figure 5:**
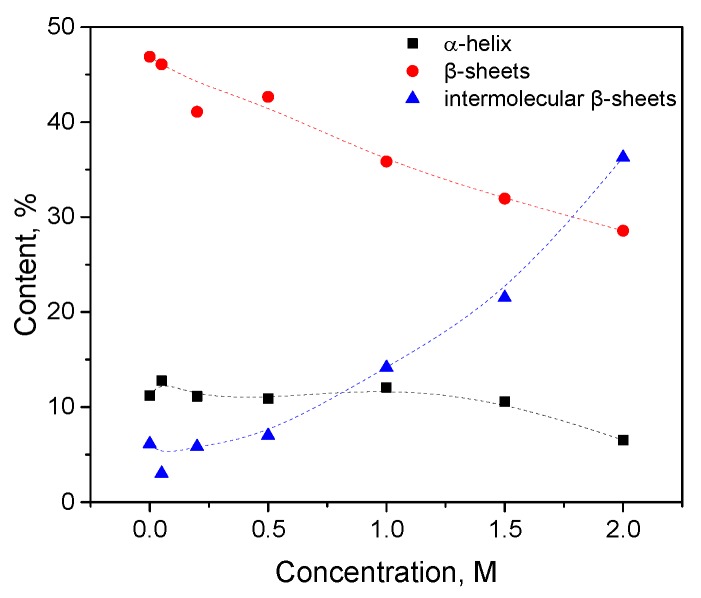
Potassium thiocyanate-induced conformational transition of trypsin at 23 °C. Protein concentration was 20% wt.

Trypsin in the same KSCN solutions undergoes intramolecular β-sheets → intermolecular β-sheets transition. As seen in Figure 4a, the maximum of the Amide I region shifts from 1638 cm^−1^ in the native form to 1633 cm^−1^ in a 2 M KSCN solution with an increase in the concentration of KSCN, suggesting a decrease of β-sheet content. At the same time, the second derivative of Amide I significantly increases the area of 1615-1625 cm^−1^, which indicates a large increase in intermolecular β-sheets, (Figure 4b). The increase in intermolecular β-sheets is significant reaching 36% in a 2 M KSCN solution (Figure 5). This is much greater than that of HSA at the same salt concentrations (Figure 5). In addition, some decrease in the α-helix content is observed in the 2 M KSCN solution, as evidenced by a decrease in the maximum in 1650–1660 cm^–1^ (Figure 4b).

**Figure 6:**
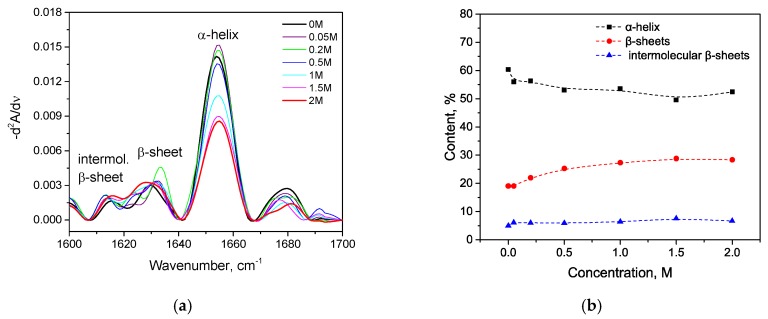
Changes in the second derivative of the Amide I band (**a**) and in secondary structure (**b**) of HSA in 10% wt. aqueous solution at 23 °C with various concentrations of KCl.

**Figure 7:**
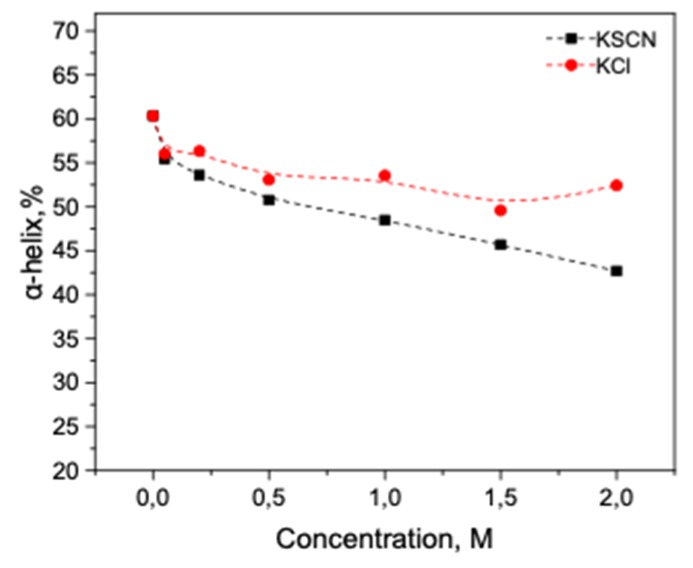
Salt-specific effects on the α-helical secondary structure of HSA measured by the FTIR spectroscopy.

KCl as a weak kosmotrope has a similar KSCN effect but to a lesser degree for both proteins. For has, we observed only an α-helix→ β-sheets transition. As can be seen in Figure 6a, peaks at the region of 1620–1640 cm^−1^ grow with an increase of the concentration of KCl, but less intensely than in the series with KSCN. The second derivatives of Amide I (Figure 6a) show a decrease in the contribution of 1650–1660 cm^–1^, which indicates a decrease in α-helicity with an increase in KCl concentration, but to a lesser extent than with the corresponding KSCN concentrations. The β-sheet content increases slightly (Figure 6b). There is no significant increase in the content of intermolecular β-sheets, as in the series with KSCN (Figure 7).

**Figure 8:**
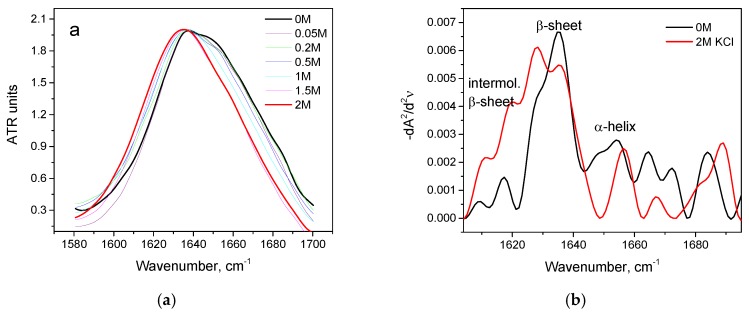
Changes in the Amide I band (**a**) and changes in the second derivative of the Amide I band (**b**) of trypsin in 20% wt. aqueous solution at 23 °C with various concentrations of KCl. Only the most representative curves are shown for the sake of clearness.

For trypsin, with the increase of KCl concentration, the maximum of the Amide I region shifts from 1638 cm^−1^ in the native form to 1634 cm^−1^ in 2 M KCl (Figure 8a) and the area of 1615–1625 cm^−1^ increases. This suggests a decrease in the intramolecular β-sheet content and an increase in intermolecular β-sheets content (Figure 8b).

**Figure 9:**
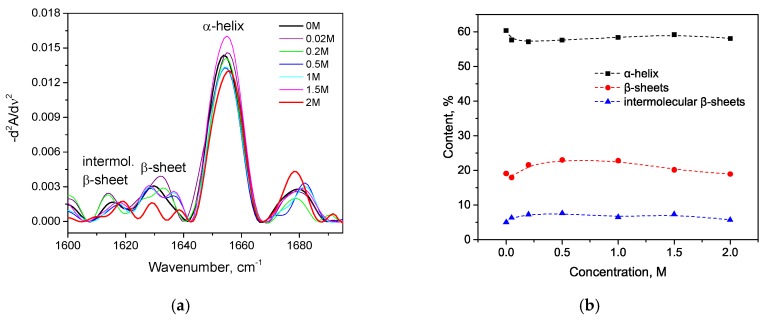
Changes in the second derivative of the Amide I band (**a**) and in secondary structure (**b**) of HSA in 10% wt. aqueous solution at 23 °C with various concentrations of (NH_4_)_2_SO_4_.

Ammonium sulfate proved to be a stabilizer. As seen in Figure 9a, there is practically no change in the Amide I region of HSA at concentrations below 1.5 M (NH_4_)_2_SO_4_. In the second derivative curves, a slight decrease in helicity was recorded (2.5% at low concentrations of up to 0.5 M and by 1% at large concentration). The content of β-sheets and intermolecular β-sheets are relatively stable in a wide range of concentrations of this salt (Figure 9b).

**Figure 10:**
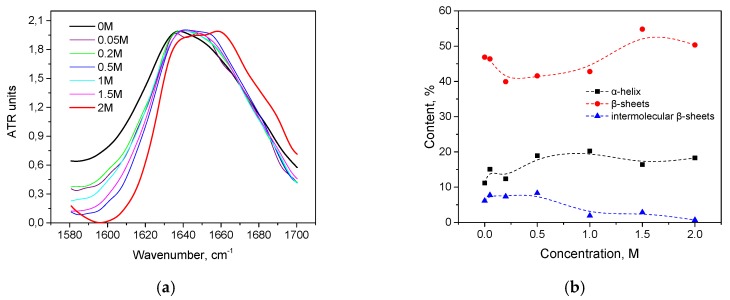
Changes in the character of the Amide I band (**a**) of trypsin at 23 °C with various concentrations of (NH_4_)_2_SO_4_ and ammonium sulfate-induced conformational transition of trypsin at 23 °C (**b**). Protein concentration was 20% wt.

Ammonium sulfate also stabilizes the trypsin structure. Figure 10a shows that with the increase of (NH_4_)_2_SO_4_ concentration there is no shift to smaller wavenumbers. The percentage of intermolecular β-sheets of trypsin significantly decreases while the contents of β-sheets (Figure 10b) and α-helices remains almost the same.

### 3.3. Combination of Salt and Temperature-Induced Transition

**Figure 11:**
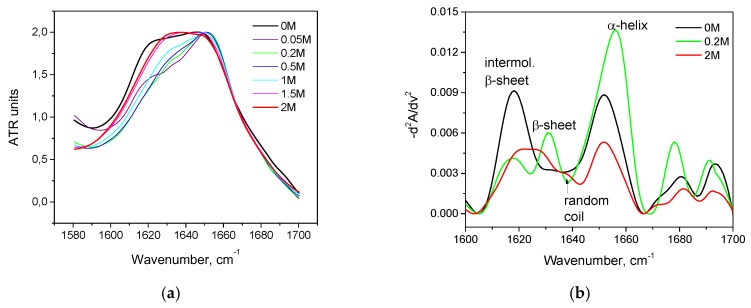
Changes in the character of the Amide I band (**a**) and changes in the second derivative of the Amide I band (**b**) of HSA in 10% wt. aqueous solution at 80 °C with various concentrations of KSCN. Only the most representative curves are shown for the sake of clearness.

**Figure 12:**
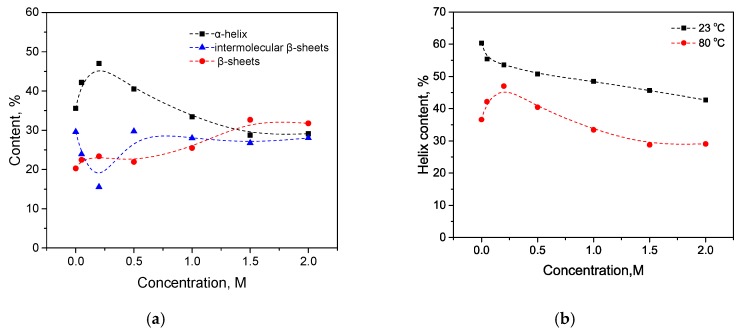
Change in the secondary structure of HSA in 10% wt. aqueous solution in the interaction with potassium thiocyanate at 80 °C (**a**) and α-helix content changes at 23 and 80 °C (**b**).

HSA is known to form intermolecular β-sheets during thermal denaturation [44,45,46,47,48], which was also observed by the FTIR [49]. In the present work, the sample was kept at a temperature of 80 C, and then the IR spectrum was immediately recorded. As seen in Figure 11a, for small concentrations of KSCN (up to 0.2 M) compared with the native form, there is no significant growth in the 1620–1640 cm^−1^ region, and there are no significant losses in the 1650–1660 cm^−1^ region. In the second derivatives, it can be seen that the concentration of 0.2 M KSCN corresponds to the largest contribution to the region of 1650–1660 cm^–1^ and the smallest contribution to the region of 1620–1640 cm^–1^ (Figure 11b). At this concentration, the largest proportion of α-helix (47%) and the smallest content of intermolecular β-sheets (15%) are observed. Moreover, at 0.2 M, the smallest relative decrease in helicity is observed (Figure 12b). With a further increase in the KSCN concentration, there is a sharp increase in the proportion of intermolecular β-sheets and a decrease in α-helicity to values lower than that of the native form (Figure 12a). This suggests that α-helices may be stabilized due to electrostatic screening at low concentrations of KSCN (up to 0.5 M) [9,10]. A further increase in KSCN concentrations led to an even greater decrease in α-helicity, which can be explained by the appearance of an ion-specific chaotropic effect. It is in line with the previous observation that chaotropic ions at low concentrations increase thermal stability for bovine serum albumin better than kosmotropic ions [44].

**Figure 13:**
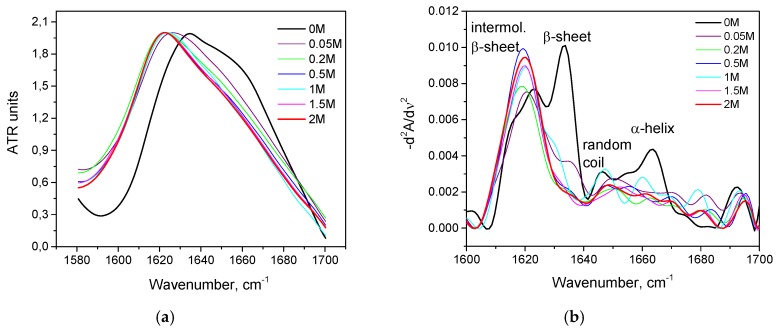
Changes in the character of the Amide I band (**a**) and changes in the second derivative of the Amide I band (**b**) of trypsin in 20% wt. aqueous solution at 80 °C with various concentrations of KSCN.

**Figure 14:**
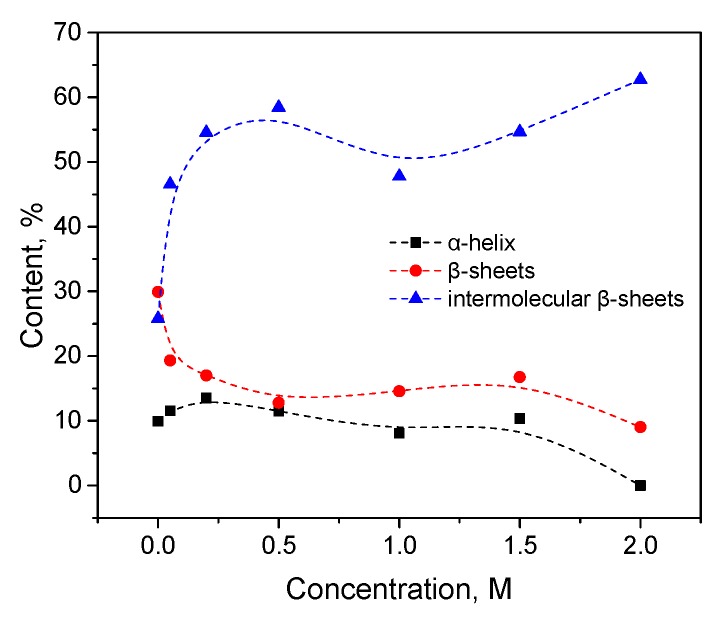
Change in the secondary structure of trypsin in the interaction with potassium thiocyanate at 80 °C.

Trypsin showed no stabilizing effects at low SCN^−^ concentration, the maximum of the Amide I region shifts to 1622 cm^−1^ at concentrations above 0.05 M, suggesting that intermolecular β-sheets became the prevailing elements in the secondary structure of trypsin (Figure 13a). Interestingly, in the area of 1615-1625 cm^−1^ of the intermolecular β-sheets of the second derivatives, its relative area at these concentrations is more than 50% (Figure 13b), while the native structure has only 26% of the intermolecular β-sheets Figure 14 shows that the addition of even a small amount of KSCN (0.05 M) leads to a significant increase in intermolecular β-sheets and reduction of intramolecular β-sheets. The α-helix content is not observed in the 2 M KSCN solution of trypsin.

**Figure 15:**
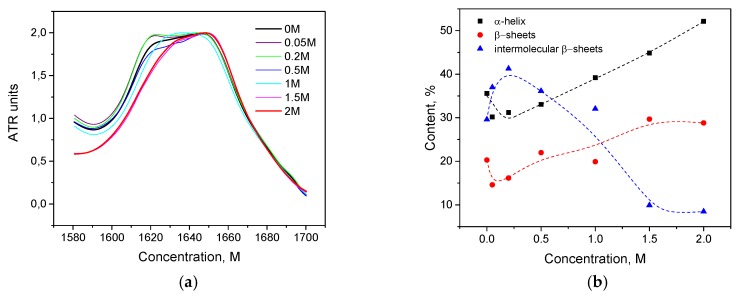
Changes in the character of the Amide I band (**a**) and conformational transition (**b**) of HSA in 10% wt. aqueous solution at 80 °C in the interaction with various concentrations of KCl.

**Figure 16:**
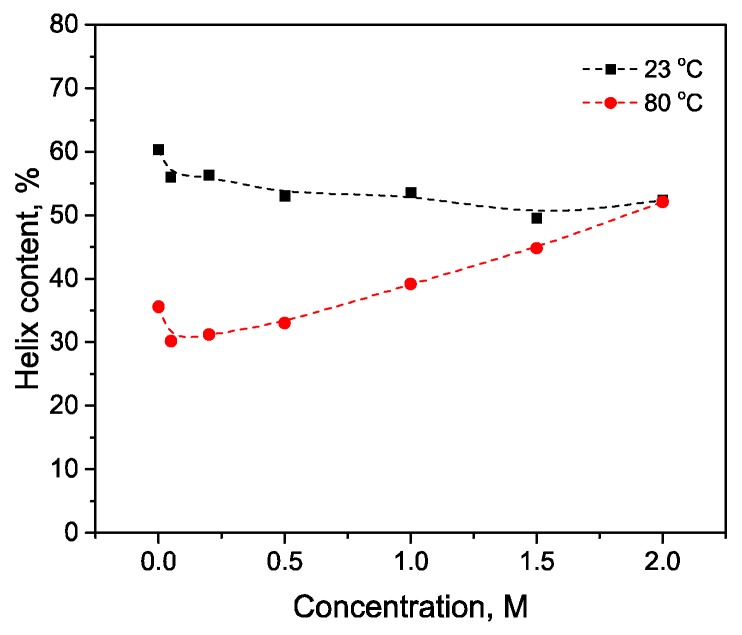
Potassium chloride-induced conformational transition of HSA at 23 °C and 80 °C.

As seen in Figure 15a, after 10 min of exposure of HSA at 80 °C and small concentrations of KCl (up to 0.5 M) an additional increase in the 1615-1625 cm^−1^ region is observed compared with the case of protein without the salt. This indicates a greater increase in intermolecular β-sheets and a greater decrease in α-helicity in comparison with the lack of KCl (Figure 15b). A further increase in the KCl concentration leads to an increase of the 1650–1660 cm^–1^ area, which can be interpreted as a restoration of the native content of α-helices. The increase in the intermolecular β-sheets content is insignificant at a concentration of 1.5 M and higher (Figure 15b). Figure 16 shows that α-helix content reduction is significantly reduced with increasing KCl concentration.

At 80 °C, KCl has a similar effect on trypsin: the growth of intermolecular β sheets which is accompanied by a decrease of intramolecular β-sheets.

**Figure 17:**
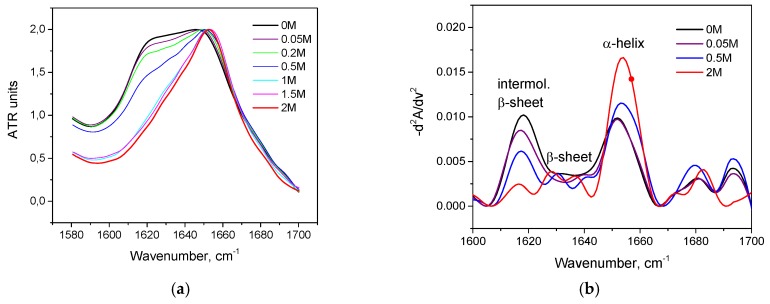
Changes in the character of the Amide I band (**a**) and changes in the second derivative of the Amide I band (**b**) of HSA in 10% wt. aqueous solution at 80 °C with various concentrations of (NH_4_)_2_SO_4_. Only the most representative curves are shown for the sake of clearness.

**Figure 18:**
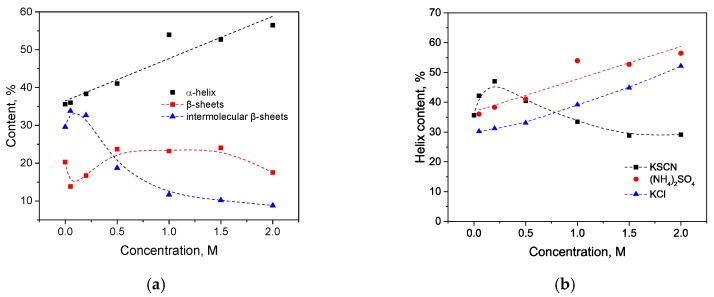
Changes in the secondary structure of HSA in the interaction with (NH_4_)_2_SO_4_ (**a**) and salt-specific effects on the α-helical secondary structure of HSA(**b**) in 10% wt. aqueous solution at 80 °C.

Figure 17a shows that with an increase (NH_4_)_2_SO_4_ concentration the region of intramolecular β-sheets significantly decreases, the α-helix increases (Figure 17b). Note that even in the low concentration of (NH_4_)_2_SO_4_, an increase in α-helicity is observed with respect to the native form, in contrast to KCl (Figure 18). (NH_4_)_2_SO_4_ exhibits kosmotropic properties, which was also described in previous studies [1,7].

**Figure 19:**
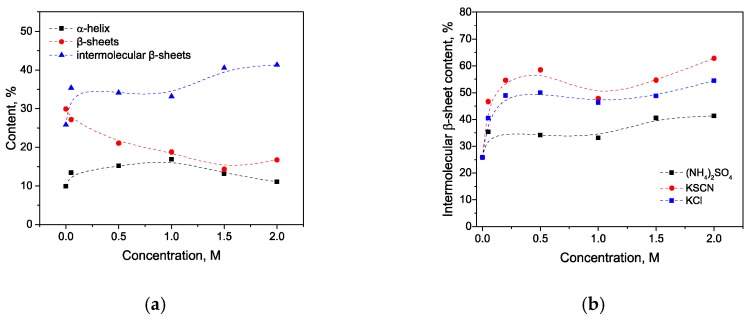
Changes in the secondary structure of trypsin depending on (NH_4_)_2_SO_4_ concentration (**a**) and salt-specific effects on intermolecular β-sheets of trypsin (**b**) in 20% wt. aqueous solution at 80 °C.

After a 10-min exposure at 80 °C in the solution of (NH_4_)_2_SO_4_, the maximum of the Amide I region shifts by 1622 cm^−1^ for all concentrations towards the region of intermolecular β-sheets, which become the prevailing elements in the secondary structure of trypsin (Figure 19a). However, this effect was smaller than in the case of KSCN and KCl (Figure 19b).

## 4. Discussion

In this study, we have examined the effect of neutral salts KSCN, KCl, and (NH_4_)_2_SO_4_ on two well-characterized proteins, α-helical human serum albumin, and β-structural porcine trypsin. Our results demonstrate that the ability of the salts to stabilize/destabilize these two proteins correlates with the Hofmeister series of ions. As the Hofmeister series predicts, KSCN will have a more chaotropic effect on proteins than KCl, and kosmotropic (NH_4_)_2_SO_4_.

The mechanism of interaction of salts with proteins is described by the LMVA theory. According to LMWA [17,18] and results of molecular dynamic and binding modeling [20,21] thiocyanate is bound to the positively charged side chain of proteins in larger quantities than chloride. It is also less hydrated than chloride and exhibits more chaotropic properties [7,17,18]. It destabilizes the native form of HSA and accumulates on the protein surface [1,42]. The binding of ions will increase the electrostatic repulsion force, which will reduce the stability of the protein and will not allow it to aggregate [6,7,17,18,31], which corresponds to the concept of absorption, as an increase in protein solubility [7,17,18]. Dynamic Light Scattering and Potentiometric titration showed that the binding of ions to bovine serum albumin (BSA) (which is a homolog of HSA) follows a Hofmeister series [21]. Thus, chloride binds to BSA weaker than thiocyanate and respectively, thiocyanate destabilizes BSA stronger than chloride. This was observed in the decrease in the melting temperature of BSA. [44]. SO_4_^−2^ increased the melting temperature and normalized the relative mutual diffusion coefficient of BSA solutions [21]. Thus (NH_4_)_2_SO_4_ is a stabilizing agent which does not bind to proteins [20,21].

In line with the previous studies, our results show that the interaction of proteins with thiocyanate and chloride ions leads to destabilization of protein structures, which manifests in the changes in the secondary structures. The processes of destabilization of both α-helical albumin and β-structural trypsin upon interaction with neutral salts lead to the formation of intermolecular β-sheets. However, due to the difference in the native structures of these proteins, their destabilization proceeds differently. For HSA with 60% of α-helical conformation, first, we observed the decrease of α-helical content with the simultaneous increase of intramolecular β-structures upon an increase of chaotropic salt (KSCN) concentration. Second, these β-structures are converted into intermolecular β-sheets. In the case of β-structural trypsin (46% of β-sheets), first, we observed the transition of intramolecular β-sheets to intermolecular ones upon an increase of KSCN concentration. The number of intermolecular β-sheets for trypsin increases 6.3 times and for albumin 3.3 times.

With a decrease in the strength of the chaotropic agents, albumin and trypsin behave similarly, however, the conformational transformations are less pronounced. For example, in the case of KCl, the α-helical content of HSA decreases by only 13%, and the intermolecular β-sheets increase by 40% of the initial values. The number of intermolecular β-sheets increases 1.26 times in albumin and 3 times in trypsin. In both cases (KSCN and KCl), trypsin aggregates stronger than albumin. This difference can be explained by a higher concentration of the trypsin solution in comparison with the HSA solution. The other explanation is that trypsin, being a more positively charged at pH 7 (pI 10.8) than albumin (pI 4.8), interacts stronger with anions of chaotropic salts.

A kosmotropic salt (NH_4_)_2_SO_4_ stabilizes the native secondary structure of albumin and trypsin. Both proteins form intermolecular *β*-sheets at high protein concentrations, but (NH_4_)_2_SO_4_ slightly decreases their percentage in comparison with KSCN and KCl.

The stabilization effect and the preservation of the secondary structure of the studied proteins with ammonium sulfate were demonstrated in the experiments at 23 °C and with heating a protein solution to a temperature above the temperature of denaturation of these proteins (80 °C). The authors of [46] using temperature-dependent AFM micrographs determined that at elevated temperatures (70 °C) albumin undergoes self-assembly with the formation of fibrillar structures. This morphological transition to form fibrils has been ascribed to the loss of α-helical content of the protein which in turn gives rise to β-sheet structures. Indeed, as can be seen from our results [31], thermal protein denaturation is accompanied by an increase in the structures of the β-sheet and intermolecular β-sheet. However, thermal denaturation processes are slowed down by an increase in the concentration of kosmotropic salt, which is manifested in a decrease in the proportion of intermolecular ß-sheets and the random coil of proteins heated to the temperature of their denaturation. Differences in the process of thermal stabilization by the KSCN and KCl salts are also explained by the LMVA theory. The value on the scale of chao–kosmotropic activity for KCl is –11.3 kJ * mol * kg^−1^ [23]. This suggests that this salt is a weak kosmotrope. As the results show, the effect of thermal stabilization is different from the effect of KSCN, and higher salt concentrations correspond to greater preservation of the HSA α-helicity. This effect was also observed in the works on the thermostability of BSA by DSC [44]. Thus, KCl occupies an intermediate position in relation to HSA, destabilizing the native form at high concentrations as a chaotropic agent, but thermostabilizing it as a kosmotopic agent. The growth of intermolecular β-sheets for trypsin was significantly higher than for HSA, which correlates with the higher amyloidogenic potential of trypsin than of HSA (Figure A1).

Numerous studies have shown [46,49,50] that under destabilizing conditions most proteins, independently of their native conformation, aggregate in the intermolecular β-structures. These β-structural aggregates have IR bands with lower wavenumbers in comparison with β-sheets of proteins in solution (for example, β-sheet of native albumin, which has a band at 1630 cm^−1^ and trypsin at 1635 cm^−1^). Amorphous aggregates that are formed upon heat denaturation usually show a strong IR band in the 1620 cm^−1^ region [46]. We also observe this band of 1620 cm^–1^ upon thermal denaturation of native albumin and trypsin. A typical band of intermolecular β-sheet of amorphous aggregates is between 1624 and 1630 cm^−1^ [46]. Amyloid fibrils, unlike amorphous aggregates, have a band at slightly higher wavenumbers (1628–1632 cm^–1^) [46]. As our results show, the interaction of albumin and trypsin with chaotropic salts leads to the formation of intermolecular β-sheets at wavenumbers of 1628 cm^−1^ [46]. This may indicate the formation of the amyloid structures; however, additional electron-microscopy and ThT-binding studies are required to confirm this conclusion.

## 5. Conclusions

The FTIR spectroscopic method was applied to the quantitative study of conformational changes in HSA and trypsin in solutions of neutral salts. The change in the secondary structure was determined by the second derivative deconvolution method of the IR spectra. Various conformational transitions were observed depending on the protein and added salt. KSCN and KCl contributed to the secondary structure transition α-helix → β-sheet of HSA. In 2 M KSCN solution α-helicity decreased 1.4 times and the β-sheet part increased 1.4 times. Intermolecular β-sheets were formed only at high concentrations of KSCN and reached 17.5%, which is 3.5 times more than in the native form of HSA. KCl had a smaller destabilizing effect, reducing the helicity by 1.15 times at 2 M. Similarly, to HSA, KSCN, and KCl contributed to the formation of non-native intermolecular β-sheets in originally β-structural trypsin. 2 M KSCN reduced the proportion of β-sheets by 1.6 times, and 2 M KCl 1.12 times. The intermolecular β-sheets formed by HSA and trypsin when interacting with chaotropic salts (KSCN and KCl) most likely correspond to the cross-β fibrillar structure. Ammonium sulfate showed a different effect on both HSA and trypsin, reducing the content of intermolecular β-sheets.

During thermal denaturation, KSCN stabilized the HSA structure at concentrations up to 0.5 M. The maximum effect was reached at 0.2 M of KSCN (α-helicity decreased by 13%, while for the native form this value is 25%). Potassium chloride and ammonium sulfate also stabilized the HSA structure. It strongly preserves HSA helicity at high concentrations (in 2 M KCl and (NH_4_)_2_SO_4_ solutions, the helicity loss was 8 and 4%, respectively). In contrast, none of the salts stabilized trypsin, moreover, 2 M KSCN had the most destabilizing effect by reducing the content of β-sheets to 9%, which is 3.3 times less than that of protein-denatured salt-free solution. The percentage of intermolecular β-sheets reached 62.7%. Potassium chloride showed intermediate chaotropic-kosmotropic properties, destabilizing the α-helical structure of HSA with increasing concentration, but also prevented thermal denaturation at high concentration. This indicates the dualism of the chloride ion effect on the human serum albumin structure.

Even though our results are mostly consistent with the effect of the salts on protein conformation predicted by the Hofmeister series, we also observed some exceptions. For example, when the thermal stability of proteins was studied in the presence of ammonium sulfate, we showed that albumin and trypsin react differently. As expected, the kosmotropic salt (NH_4_)_2_SO_4_, significantly stabilized the secondary structure of albumin when heated to 80 °C, however, surprisingly, it did not have a stabilizing effect on trypsin, increasing the content of intermolecular β-sheets upon the increase of the salt concentration.

Thus, in this work, FTIR spectroscopy made possible the stepwise evaluation of the salt-dependent conformational changes in proteins. It was possible to quantitatively trace the transition of α-helices to β-structures in the analyzed proteins. With an increase in the concentration of chaotropic (according to the Hofmeister series) salts, these proteins were transformed into the aggregates with intermolecular β-sheets. In addition, FTIR spectroscopy data allowed us to conclude that these aggregates have fibrillar or amorphous structures. Such a detailed analysis of the salt effects may further clarify the mechanism of formation of amyloid fibrils and amorphous protein aggregates, as well as create strategies preventing the misfolding of proteins.

## Figures and Tables

**Table 1:** Properties of the ions according to “law of matching water affinities” (LMWA) [7,17,18].

Type	Ions	Jones–Dole Viscosity B Coefficient	Specific Effect on Water	Specific Effect on Protein
Cations	K^+^	−0.007	Chaotrope	stabilization
	NH_4_^+^	−0.007	Chaotrope	stabilization
Anions	SCN^−^	−0.103	Chaotrope	denaturation
	Cl^−^	−0.007	Chaotrope	denaturation
	SO_4_^2−^	0.208	Kosmotrope	stabilization

**Table 2:** The correlation between protein secondary structure and the second derivative of Amide I [32,33].

Wavenumber, cm^−1^	Range, cm^−1^	Secondary Structure
1618	1610–1630	Intermolecular β-sheet
1628,1638,1691	1630–1639, 1689–1695	β-sheet
1650, 1657	1650–1660	α-helix
1673, 1681	1660–1689	β-turn

**Table 3:** The HSA and trypsin secondary structure.

Protein	Data Source	β-Turn, %	α-Helix, %	Irregular Structure, %	β-Sheet, %
HSA	DSSP [36]	8.9	68.4	12.9	-
	STRIDE [39]	15.7	69.0	12.5	-
	X-ray [34]	2.1	68.8	21.8	7.3
Trypsin	DSSP [36]	14.8	10.3	30.0	32.3
	STRIDE [39]	36.7	10.8	19.7	32.7
	X-ray [35]	4.7	9.9	41.9	43.3

## Supplementary Materials

The supplementary materials are available online at https://www.mdpi.com/2218-273X/10/4/606/s1.
