# Supplementary material for: FTIR Spectroscopy Study of the Secondary Structure Changes in Human Serum Albumin and Trypsin under Neutral Salts
Source: Biomolecules. 2020 Apr 14;10(4):606. doi: 10.3390/biom10040606 (PMC7226448; doi:10.3390/biom10040606)
Supplement: Supplementary file 1 [file biomolecules-10-00606-s001.pdf]

Supplemntary Figures

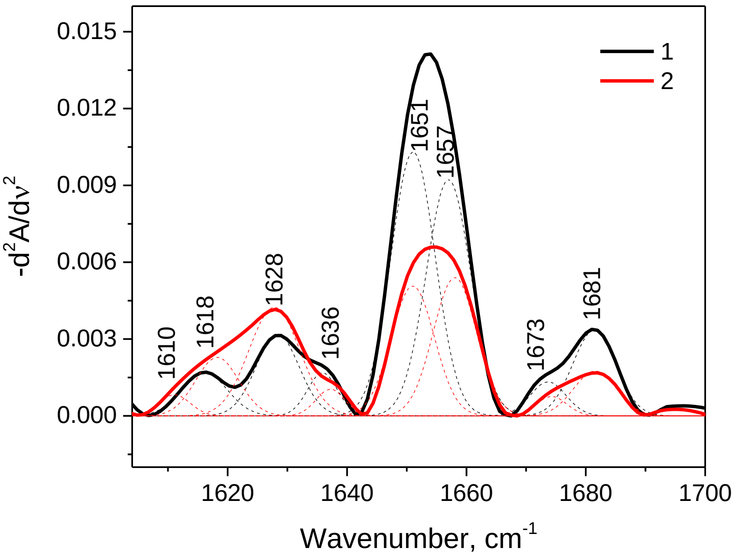

Figure S1. Deconvolution of the second derivative of the HSA spectrum in native form (1) and in a 2 M solution of KSCN (2).

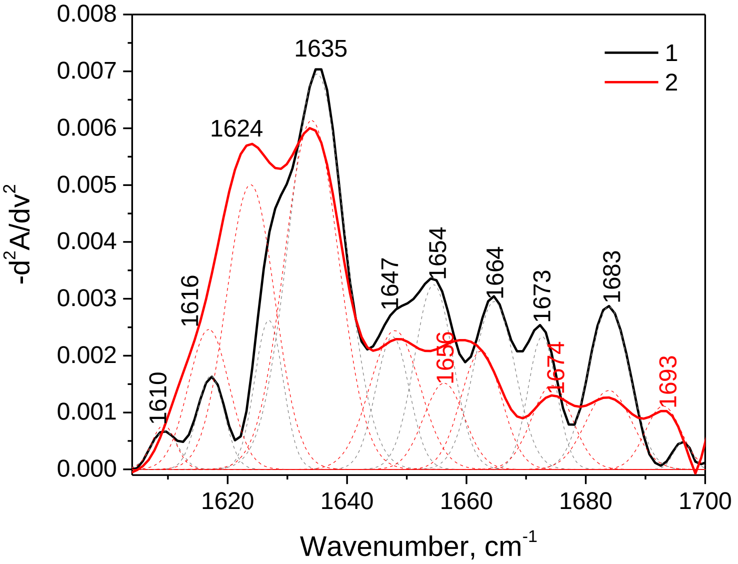

Figure S2. Deconvolution of the second derivative of the trypsin spectrum in native form (1) and in a 2 M solution of KSCN (2).

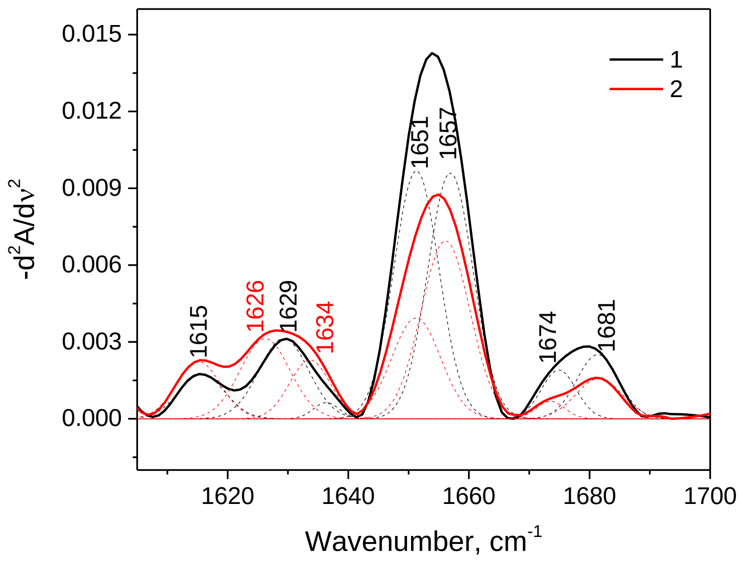

Figure S3. Deconvolution of the second derivative of the HSA spectrum in native form (1) and in a 2 M solution of KCl (2).

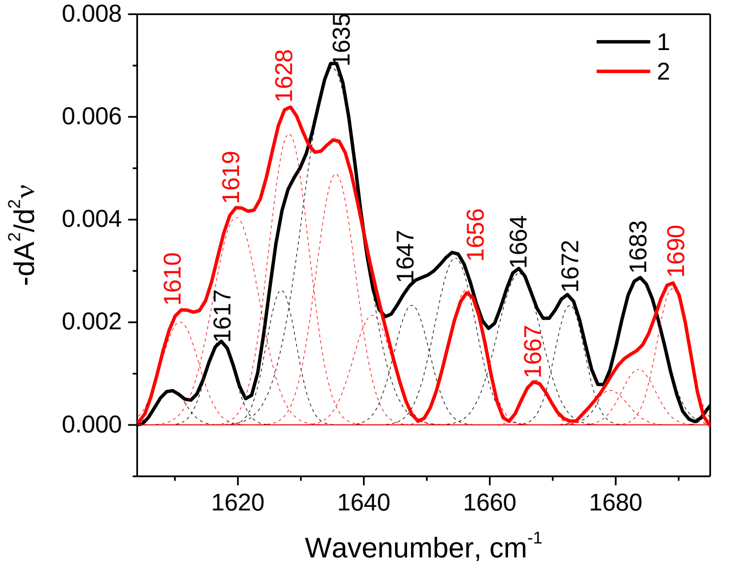

Figure S4. Deconvolution of the second derivative of the trypsin spectrum in native form (1) and in a 2 M solution of KCl (2).

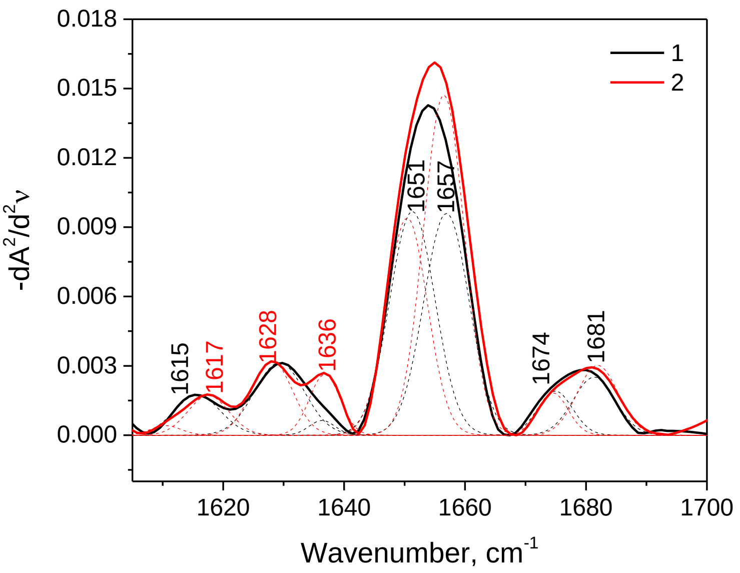

Figure S5. Deconvolution of the second derivative of the HSA spectrum in native form (1) and in a 2 M solution of $(NH_4)_2SO_4$ (2).

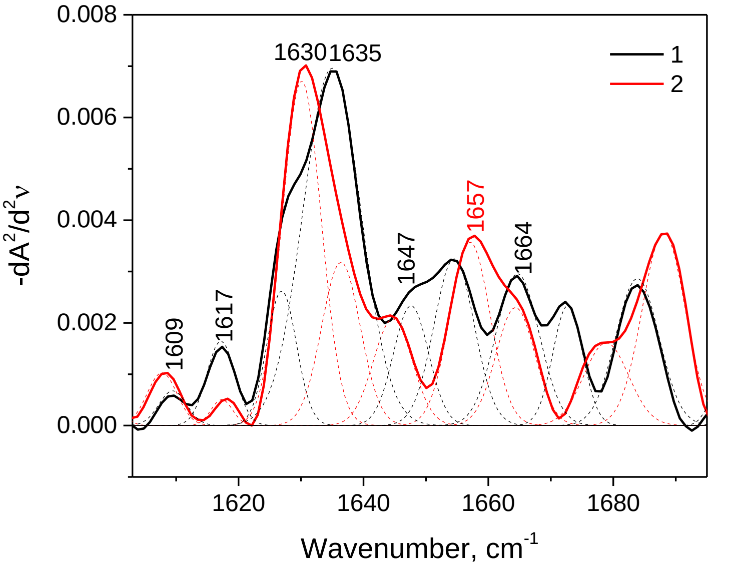

Figure S6. Deconvolution of the second derivative of the trypsin spectrum in native form (1) and in a 2 M solution of $(NH_4)_2SO_4$ (2).
